# Adenosine/A_2A_R/PKA signaling regulates HO-1-mediated anti-inflammatory responses during *Leishmania donovani* infection

**DOI:** 10.1128/mbio.02581-25

**Published:** 2025-11-24

**Authors:** Tapasi Das, Pronay Brahmachari, Anindita Ukil

**Affiliations:** 1 Department of Biochemistry, University of Calcutta 30163, Kolkata, West Bengal, India; University of Geneva, Geneva, Switzerland

**Keywords:** adenosine receptor, PKA, *Leishmania donovani*, macrophage, heme oxygenase-1

## Abstract

Adenosine receptor A_2A_R plays a pivotal role in dampening pro-inflammatory cytokine levels in *Leishmania donovani*-infected macrophages, thus promoting infection. However, the specific regulatory pathway remains unidentified. In this study, we showed that blocking A_2A_R signaling reduces the expression of heme oxygenase-1 (HO-1), an enzyme earlier implicated in reducing pro-inflammatory cytokine levels. A_2A_R, being a G-protein-coupled receptor (GPCR), increased intracellular cAMP, thereby activating protein kinase A (PKA) activity. Inhibition of the A_2A_R/PKA pathway impacted two major transcription factors of HO-1, cAMP response element-binding protein (CREB) and nuclear factor erythroid 2-related factor 2 (NRF2). PKA directly activated CREB through phosphorylation, and the ChIP assay further validated the involvement of PKA in p-CREB-mediated HO-1 transcription. On the other hand, PKA-mediated glycogen synthase kinase-3 beta (GSK-3β) phosphorylation at the Ser-9 position rendered it inactive and removed its inhibitory effect on NRF2, thus allowing its nuclear translocation during infection. Macrophages transfected with constitutively active nonphosphorylated GSK-3β showed reduced nuclear localization of NRF2 and decreased parasite survival. Administering the A_2A_R inhibitor in infected mice decreased HO-1 levels, liver and spleen parasite burden, and increased pro-inflammatory cytokine levels. Our findings revealed *Leishmania* exploits adenosine-A_2A_R signaling to activate PKA-mediated CREB- and NRF2-dependent HO-1 upregulation, reducing pro-inflammatory cytokine levels and favoring pathogenesis.

**IMPORTANCE:**

Visceral leishmaniasis, caused by the protozoan parasite *Leishmania donovani*, is a major health concern affecting over a million people worldwide. An increase in host ATP production and its efflux benefits the survival of Leishmania parasites and prolongs the infection. Effluxed ATP is converted to adenosine, which activates adenosine-A_2A_R signaling to provide an immunosuppressive milieu, necessary for infection propagation. This study identified cAMP/PKA as the essential components of A_2A_R signaling, which further differentially activate two transcription factors to induce the antioxidant enzyme HO-1, responsible for creating the anti-inflammatory environment. Our findings highlight A_2A_R as a promising drug target against visceral leishmaniasis and other inflammation-related diseases, offering us the opportunity to alleviate inflammatory responses, thereby broadening the impact on disease management and therapy.

## INTRODUCTION

Visceral leishmaniasis (VL), commonly known as kala-azar, is the most acute form of leishmaniasis, which is caused by the intracellular protozoan parasite *Leishmania donovani* (1). VL primarily affects internal organs such as the liver, spleen, and bone marrow and manifests with systemic infection characterized by prolonged fever, hepatosplenomegaly, weight loss, anemia, and immunosuppression (2). Currently available medications are few and often found to be associated with toxicity (3). Increasingly emerging cases of drug resistance in endemic regions further complicate the scenario and highlight the urgent need for improved therapeutic and preventive strategies (4, 5).

The pervasiveness of VL is greatly influenced by the successful outcome of macrophage–*Leishmania* co-inhabitance (6), where the parasite often manipulates the immune-metabolic profile of the host and turns it into a pathogen-permissive repository (7). Increasing the host’s energy yield for the benefit of the parasite is one of the important modulatory mechanisms, and *Leishmania* achieves this by inducing ATP-producing catabolic pathways, glycolysis, and fatty acid oxidation (8). ATP is utilized both as an energy currency and a source of intracellular and extracellular messenger molecules, such as cAMP and adenosine (9). Under stressful conditions, cells release ATP to the extracellular medium, where ATP-hydrolyzing enzymes, CD39 (also known as ENTPD1) (10) and CD73 (also known as NT5E) (11), sequentially convert ATP into adenosine (12). This extracellular adenosine further interacts with P1 purinergic receptors and helps in the establishment of an anti-inflammatory environment, thus protecting against tissue damage (13). Four types of adenosine purinergic receptors belonging to the GPCR superfamily have been reported: A_1_R, A_2A_R, A_2B_R, and A_3_ (14). Our previous study showed that *L. donovani* infection not only prompts macrophages to release ATP and produce extracellular adenosine but also induces the expressions of A_2A_R and A_2B_R on the cell surface. Interaction between A_2A_R and adenosine showed significantly decreased production of pro-inflammatory cytokines TNF-α and IL-12, thereby helping create a parasite-favorable environment (15).

A_2A_R has a high affinity for adenosine (16, 17) and is widely distributed on a variety of immune cell surfaces (18–20). The adenosine-A_2A_R-mediated signaling pathway is primarily dedicated to reducing pro-inflammatory cytokine levels (15) and is frequently exploited by other intracellular pathogens also (21–23). However, the detailed adenosine-mediated signaling cascade that leads to the establishment of such an environment in the context of VL has still not been studied. Heme oxygenase-1 (HO-1) is an antioxidant heme-degrading enzyme, and along with neutralizing reactive oxygen species (ROS), HO-1 disrupts TLR4 signaling in *Leishmania*-infected macrophages, thus reducing pro-inflammatory cytokine production (24). Previously, we uncovered nuclear factor erythroid 2-related factor 2 (NRF2) as one of the major transcription factors regulating HO-1 expression during infection (25), but the detailed signaling pathway that triggers HO-1 expression during VL has not yet been investigated. Since both adenosine/A_2A_R and NRF2/HO-1 signaling are activated by *Leishmania* and contribute to creating an anti-inflammatory environment, there is a possibility of an existing alliance or hierarchy between the pathways.

A_2A_R has been reported to inhibit the pentose phosphate pathway and limit ROS production during *Staphylococcus aureus* infection, increasing its survival rate (23). A_2A_R, as a stimulatory G protein-coupled receptor (GPCR), activates adenylate cyclases (ACs), which have been shown to maintain antioxidant protein homeostasis for ROS detoxification in *L. donovani-*infected host cells (26). Increased cAMP levels and subsequent downstream signaling are reported to inhibit T-cell activation and proliferation (18). The adenosine-A_2A_R signaling pathway plays a nonredundant role in not just cAMP-mediated immunosuppression but also attenuation of tissue damage (27). cAMP-mediated signaling pathways are reported in *L. donovani* pathogenesis (28), and the contribution of its downstream effector PKA in malaria and other apicomplexan parasitic diseases has also been documented (29, 30). Involvement of the PKA-HO-1 signaling pathway in lipoteichoic acid (LTA)-stimulated inflammation in BV2 microglial cells is also reported (31), and all these studies increase the possibility of the activation of a functional A_2A_R/PKA/NRF2/HO-1 axis in *Leishmania* infection.

We report here that *Leishmania*-induced A_2A_R pathway activates cAMP/PKA signaling. PKA directly phosphorylates CREB, necessary for HO-1 transcription. On the other hand, it activates NRF2, another important transcription factor of HO-1, through phosphorylation-mediated inactivation of GSK-3β. The culminated effect was pronounced HO-1 activation, resulting in an anti-inflammatory environment, essential for parasite survival.

## RESULTS

### A_2A_R activation induces HO-1 expression during *Leishmania* infection

**Fig 1:**
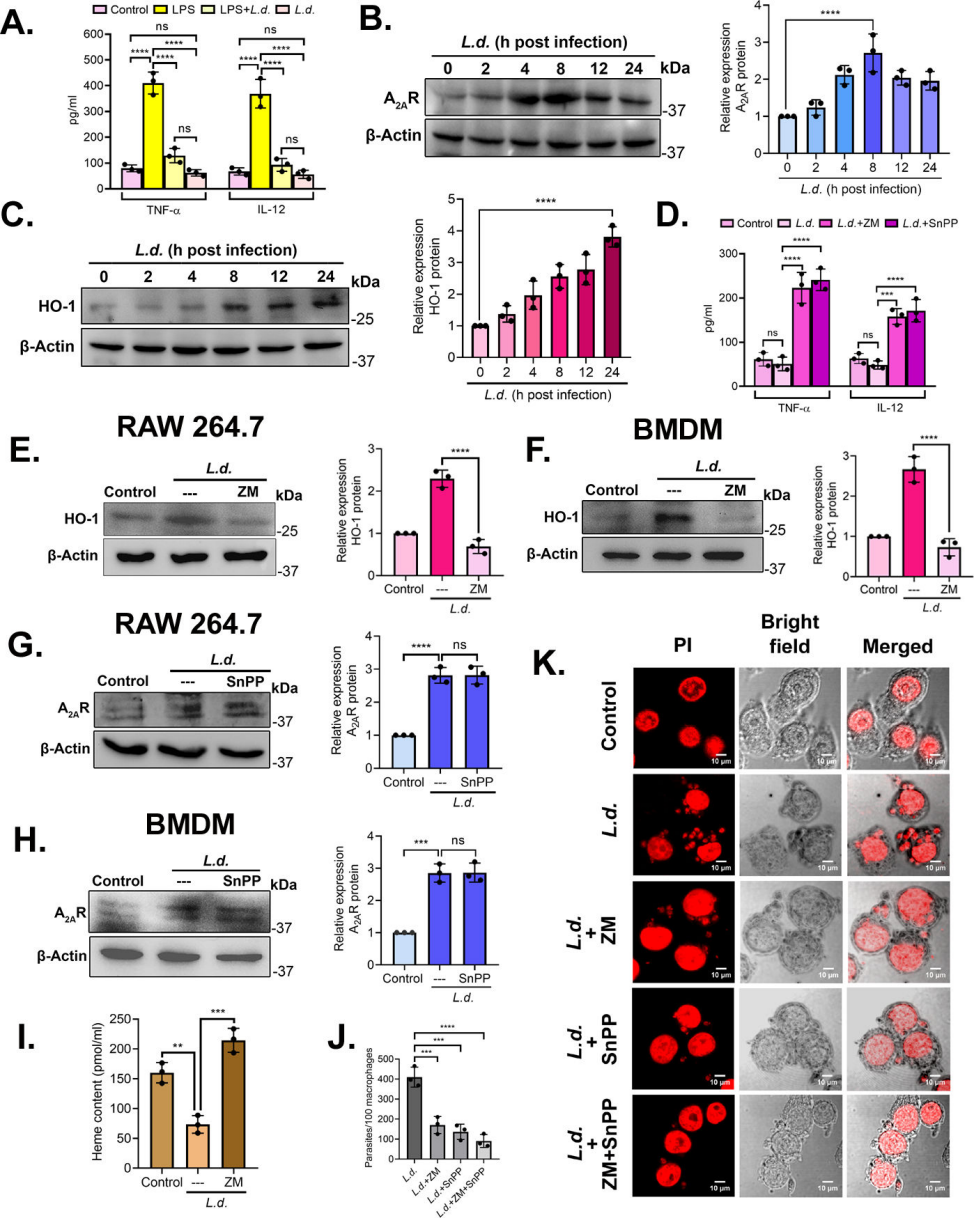
A_2A_R activation upregulates HO-1 expression in *L. donovani* infection. (**A**) RAW 264.7 cells were either treated with lipopolysaccharide (LPS) (100 ng/mL) or both LPS and *L.d* or only infected with *L. donovani* promastigotes for 24 h, and cell culture supernatants were assayed for TNF-α and IL-12 using ELISA. (**B and C**) RAW cells were infected with *L. donovani* for the indicated periods (0–24 h) and the expressions of A_2A_R and HO-1 were assayed at the protein level by immunoblotting. (**D**) RAW 264.7 cells were either treated with LPS or ZM 241385 (1 μM) or SnPP (20 µM) and infected with *L. donovani* for 8 h, and cell culture supernatants were assayed for TNF-α and IL-12 using ELISA. (**E and F**) RAW and bone marrow-derived macrophage (BMDM) cells were pretreated with ZM 241385 (1 μM) for 30 min followed by *L. donovani* infection for 8 h, and the expression of HO-1 was measured at the protein level by immunoblotting. (**G and H**) RAW and BMDM were pretreated with SnPP (20 µM) for 1 h followed by *L. donovani* infection for 8 h, and the expression of A_2A_R was measured at the protein level by immunoblotting. (**I**) RAW cells either infected with *L. donovani* for 8 h or pretreated with SnPP (20 µM), followed by infection for 8 h. Heme content was then measured in cell lysates with formic acid, and results were expressed as picomoles per mL. (**J and K**) RAW cells were either uninfected or infected with *L. donovani* promastigotes or pretreated with ZM 241385 (1 μM), SnPP (20 µM) and both, followed by infection for 8 h. The number of parasites per 100 macrophages was determined by propidium iodide staining (**J**), and representative confocal microscopic images are depicted (**K**). The graph shows the combined (mean) outcomes from the indicated number of independent experiments, and the error bars indicate the variation between those independent repeats (mean ± SD); ns, not significant, **P < 0.01, ***P < 0.001, and ****P < 0.0001 (ANOVA with Tukey *post hoc* test).

*L. donovani* strategizes its survival inside the hostile environment of macrophages by downregulating the expression of pro-inflammatory cytokines, TNF-α and IL-12 (15). Likewise, we have found that the upregulation of TNF-α and IL-12 in LPS (a positive stimulator of pro-inflammatory responses [32])-treated macrophages (410.6 ± 42.8 pg/mL for TNF-α and 368.6 ± 55.6 pg/mL for IL-12) at 24 h post infection was significantly reduced (128.8 ± 27.8 pg/mL for TNF-α and 92.9 ± 24.8 pg/mL for IL-12) in LPS +*L.d*.-treated cells (*P* < 0.0001 and *P* < 0.0001, respectively) (Fig. 1A). Two effective regulators are known to create such parasite conductive anti-inflammatory environment inside the host, A_2A_R (15) and HO-1 (24). Both the proteins documented increased expression in infected cells (2.7-fold at 8 h post-infection and 3.8-fold at 24 h post-infection over uninfected controls, for A_2A_R and HO-1, *P* < 0.0001 and *P* < 0.0001, respectively) (Fig. 1B and C). As expected, infected macrophages pretreated with A_2A_R and HO-1 specific inhibitors, ZM 241385 (1 μM) (15) and SnPP (20 µM) (24), respectively, showed significant enhancement in pro-inflammatory cytokine levels [223.1 ± 34.9 pg/mL TNF-α and 158.1 ± 17.6 pg/mL IL-12 for ZM 241385 and 241.1 ± 24.5 pg/mL TNF-α and 171.2 ± 25.2 pg/mL IL-12 for SnPP, compared with 50.8 ± 15.5 pg/mL TNF-α and 48.6 ± 9.1 pg/mL IL-12 for infected control, *P* < 0.0001 and *P* < 0.0001, respectively] (Fig. 1D). Cells pretreated with either ZM 241385 or SnPP did not document any significant change in the rate of infection (see Fig. S1A at https://doi.org/10.6084/m9.figshare.30597143). As both A_2A_R and HO-1 showed important roles in reducing inflammation and both of their expressions increased during infection, we therefore wanted to determine whether they are interrelated. Since we found maximum A_2A_R expression at 8 h post-infection, we checked the A_2A_R-HO-1 correlation at the 8 h time point. RAW 264.7 cells were first treated with ZM 241385 (1 µM) followed by *L. donovani* infection for 8 h, and HO-1 expression was evaluated. Inhibition of A_2A_R resulted in a significant decrease in HO-1 expression (70.0% reduction compared with infected control, *P* < 0.0001) (Fig. 1E). A similar reduction was found in bone marrow-derived macrophages (BMDMs) pretreated with ZM 241385 and infected for 8 h (72.6% reduction compared with infected control, *P* < 0.0001) (Fig. 1F). On the contrary, HO-1 inhibition by SnPP (20 µM) treatment did not exert any noticeable effects on A_2A_R expression at 8 h post-infection for both RAW 264.7 and BMDM (Fig. 1G and H), suggesting that A_2A_R possibly is an upstream regulator of HO-1. So far, we suggested that HO-1 expression depends on A_2A_R activation, and since HO-1 is a heme-degrading enzyme, we further checked heme content in infected ZM 241385-treated samples, and as expected, ZM 241385 reversed the infection-induced decrease in heme content (2.9-fold increase compared to infected control, *P* = 0.0002) (Fig. 1I). Both the inhibitors decreased parasite survival in RAW 264.7 cells (58.4% decrease with ZM 241385 and 66.7% decrease with SnPP, compared with infected control, at 8 h post-infection, *P* = 0.0005 and *P* = 0.0002, respectively). However, the combined inhibitor treatment caused a higher reduction of parasite burden (77.9% decrease compared with infected control at 8 h post-infection, *P* < 0.0001) (Fig. 1J and K), suggesting synergistic actions. These observations suggest that A_2A_R is one of the critical regulators of HO-1 during infection.

### A_2A_R regulates HO-1 expression through cAMP-dependent PKA during *L. donovani* infection

**Fig 2:**
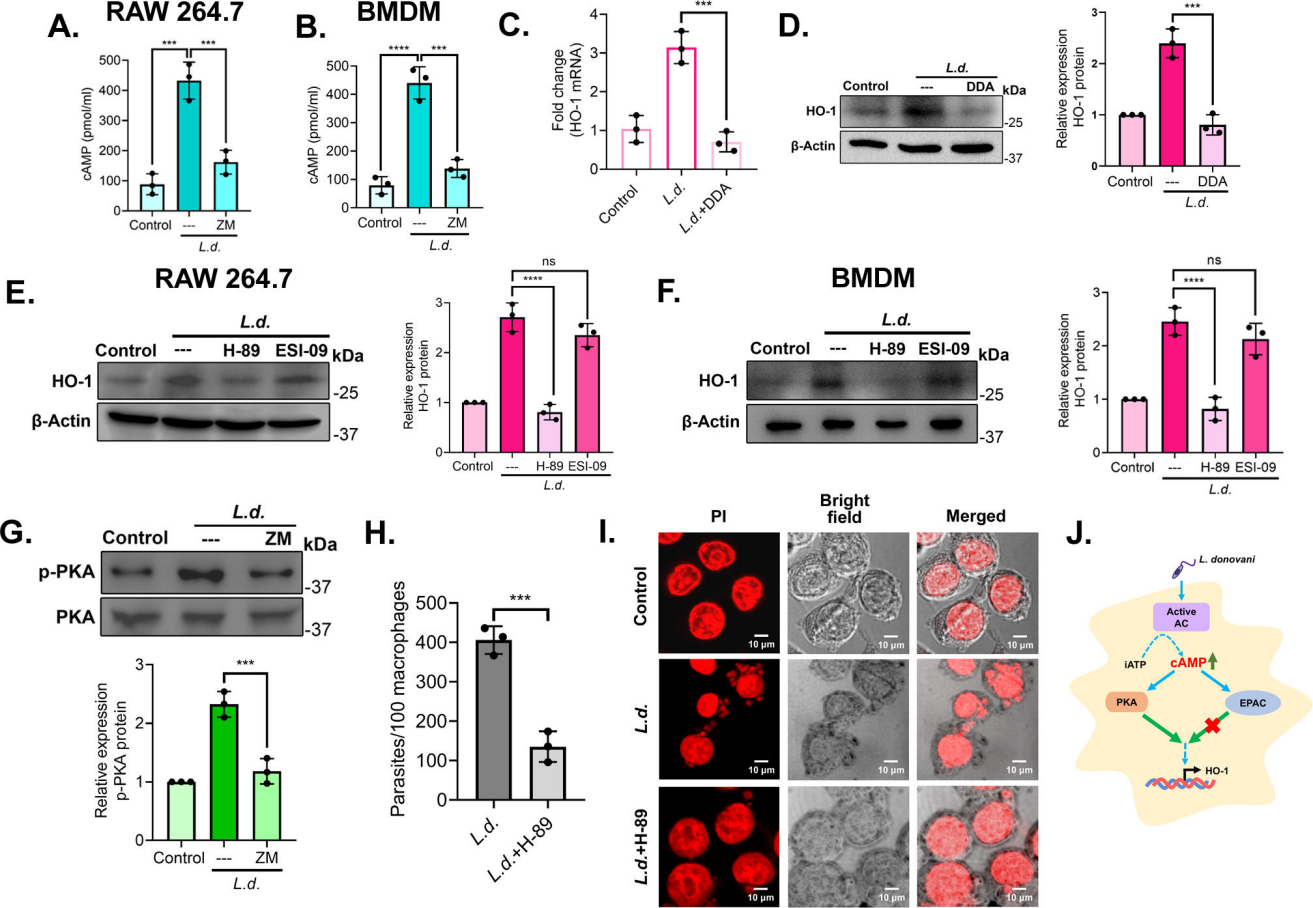
A_2A_R modulates HO-1 expression via cAMP-dependent PKA signaling during *L. donovani* infection. (**A and B**) The intracellular cAMP level was determined in ZM 241385-pretreated infected RAW 264.7 (**A**) and BMDM (**B**). (**C and D**) RAW cells were pretreated with DDA (100 µM), and the expression of HO-1 was evaluated at the mRNA level (**C**) and protein level (**D**). (**E and F**) Protein level expression of HO-1 was determined in H-89 (10 µM) and ESI-09 (10 µM)-pretreated infected RAW cells (**E**) and BMDM (**F**). (**G**) RAW cells were infected with *L. donovani* (8 h) in the presence or absence of ZM 241385 (1 μM), and the phosphorylated PKA level was evaluated by immunoblotting. (**H and I**) RAW macrophages were either uninfected or infected with *L. donovani* promastigotes or pretreated with H-89 (10 µM) followed by infection. The number of parasites per 100 macrophages was determined by PI staining (**H**), and representative confocal microscopic images are depicted (**I**). (**J**) Schematic representation of cAMP-PKA-mediated expression of HO-1 during infection. The graph shows the combined (mean) outcomes from the indicated number of independent experiments, and the error bars indicate the variation between those independent repeats (mean ± SD); ns, not significant, ***P < 0.001, ****P < 0.0001 (Student’s *t*-test and ANOVA with Tukey *post hoc* test).

As A_2A_R is a GPCR, the activation of A_2A_R in infection might result in the activation of adenylate cyclase and elevated intracellular cAMP levels. To prove this, we measured the intracellular cAMP level in uninfected control, infected, and ZM 241385-pretreated infected RAW 264.7 and BMDM at 8 h post-infection. Infection resulted in a significant increase in cAMP level (4.9-fold and 5.2-fold increase compared with uninfected control, in RAW 264.7 cells and BMDM, *P* = 0.0002 and *P* < 0.0001, respectively). However, ZM 241385 treatment led to a marked decrease in cAMP level in infected cells (62.6% and 68.5% decrease compared with infected control, in RAW 264.7 and BMDM, *P* = 0.0009 and *P* = 0.0003, respectively) (Fig. 2A and B). To evaluate the effect of intracellular cAMP levels on HO-1 expression, we used the cAMP inhibitor DDA (100 µM) (33) and checked both HO-1 mRNA and protein levels. Treatment with DDA showed a marked decrease in both HO-1 mRNA and protein levels (77.5% and 66.4% decrease compared with infected control, in case of mRNA and protein expressions, *P* = 0.0003 and *P* = 0.0002, respectively) (Fig. 2C and D). Since the effects of cAMP are primarily mediated by PKA and EPAC (34, 35), we therefore used PKA-inhibitor H-89 (10 µM) (33) and EPAC-inhibitor ESI-09 (10 µM) (33) to check their contribution toward regulating HO-1 expression. Pretreatment with H-89 notably decreased HO-1 expression (70.3% decrease compared with infected control, *P* < 0.0001) (Fig. 2E). However, there was no significant decrease in HO-1 expression by pretreatment with ESI-09. A similar observation was obtained in BMDM (66.7% decrease compared with infected control, in the case of H-89-treated cells, *P* < 0.0001) (Fig. 2F). Increasing concentration of ESI-09 also produced no impact on HO-1 expression (data not shown) and thus prompted us to exclude EPAC from subsequent experiments. All drug treatments employed in these experiments have been previously evaluated for their impact on the rate of infection, and no significant alterations were observed (see Fig. S1B at https://doi.org/10.6084/m9.figshare.30597143). To confirm the involvement of A_2A_R in PKA activation, phospho-PKA level was determined in the presence of ZM 241385, which showed a marked decrease in its activation (49.2% decrease compared with infected control, *P* = 0.0005) (Fig. 2G). These observations corroborated the role of the A_2A_R/cAMP/PKA signaling pathway in regulating HO-1 during infection. Treatment with H-89 significantly decreased parasite count in RAW 264.7 cells as well (66.7% decrease compared with infected control, *P* = 0.0009) (Fig. 2H and I). This decrease in parasite number was not due to any change in the rate of infection for H-89 pretreatment (see Fig. S1C at https://doi.org/10.6084/m9.figshare.30597143). All these results suggest that A_2A_R activation elevates intracellular cAMP level, which activates HO-1 expression through PKA (Fig. 2J).

### *L. donovani* induces PKA-mediated phosphorylation of CREB which contributes to HO-1 expression

**Fig 3:**
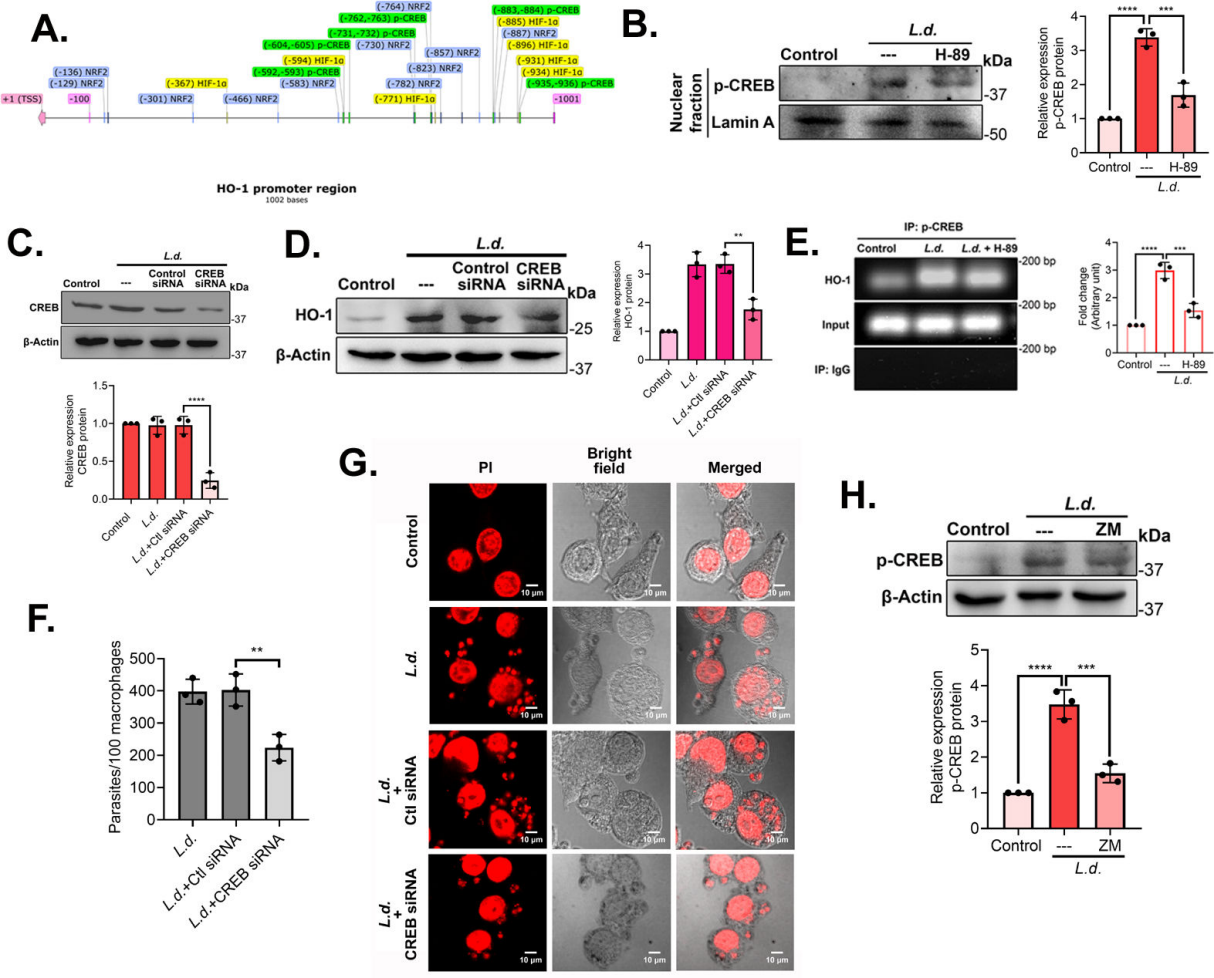
*L. donovani* triggers PKA-mediated CREB phosphorylation and HO-1 activation. (**A**) Schematic representation of p-CREB, NRF2, HIF-1α, and AP-1 binding sites in HO-1 promoter cells. (**B**) RAW cells were pretreated with H-89 (10 µM) followed by infection with *L. donovani,* and the levels of phosphorylated CREB were analyzed in nuclear fraction by immunoblotting. (**C**) Macrophages were transfected with CREB siRNA and then infected with *L. donovani,* and siRNA efficiency was evaluated by immunoblot analysis. (**D**) HO-1 protein expression was assessed in infected macrophages treated with CREB siRNA by immunoblotting. (**E**) DNA from *L. donovani*–infected RAW cells was immunoprecipitated with anti-p-CREB antibody or normal immunoglobulin G. Immunoprecipitated DNA was then analyzed using HO-1 promoter-specific primers by PCR, followed by agarose gel electrophoresis. (**F and G**) BMDMs were either uninfected or infected with *L. donovani* (8 h) in the presence or absence of CREB siRNA treatment. The number of parasites per 100 macrophages was determined by PI staining (**F**), and representative confocal microscopic images were depicted (**G**). (**H**) Infected macrophages were pretreated with ZM 241385, and the p-CREB protein level was checked by immunoblotting. The graph shows the combined (mean) outcomes from the indicated number of independent experiments, and the error bars indicate the variation between those independent repeats (mean ± SD); **P < 0.01, ***P < 0.001, and ****P < 0.0001 (ANOVA with Tukey *post hoc* test).

To get an insight into PKA-mediated HO-1 activation, promoter analysis was carried out for possible transcription factors of HO-1, and NRF2, p-CREB, and HIF-1α were identified (Fig. 3A). The promoter analysis figure was designed using SnapGene software (https://www.snapgene.com/). As HIF-1α is predominantly regulated by EPAC (33), we excluded it for the time being. Infection resulted in an increased level of p-CREB (3.4-fold increase over uninfected control, *P* < 0.0001) in the nuclear extract, and this was found to be reversed by H-89 pretreatment (50.0% decrease compared with infected cells, *P* < 0.0001) (Fig. 3B). To ascertain the role of CREB in the induction of HO-1 in infection, a siRNA-mediated knockdown system was used. CREB siRNA treatment showed a 74.9% reduction (*P* < 0.0001) of CREB expression compared to control siRNA-treated infected cells (Fig. 3C). Transfections with siRNA did not show any detectable change in the rate of infection (see Fig. S1D at https://doi.org/10.6084/m9.figshare.30597143). Protein levels of HO-1 were markedly decreased (47.4% in CREB siRNA-treated infected macrophages, compared with control siRNA-treated infected cells, *P* = 0.0015) (Fig. 3D), indicating that CREB induces HO-1 expression during infection. We also performed a chromatin immunoprecipitation (ChIP) assay to show the recruitment of p-CREB to the promoter region of HO-1 at 8 h post-infection. The results showed significantly strong binding during infection compared to the uninfected control. However, this binding was significantly decreased (48.5%, *P* = 0.0005) in H-89-treated infected macrophages (Fig. 3E). The effect of treatment with CREB siRNA was also reflected on parasite survival in BMDMs, which was significantly decreased (44.4% compared with control siRNA-treated infected cells, *P* = 0.0057) (Fig. 3F and G). Finally, to ascertain the role of A_2A_R in CREB regulation, p-CREB levels were determined in ZM 241385 pretreated infected macrophages. A significant decrease was found in p-CREB expression in inhibitor-treated cells (55.5% compared with infected control, *P* = 0.0004) (Fig. 3H). All these findings validated the A_2A_R/PKA/CREB axis in regulating HO-1 expression by facilitating PKA-mediated CREB phosphorylation.

### PKA mediates nuclear translocation of NRF2 through inhibition of GSK-3β

**Fig 4:**
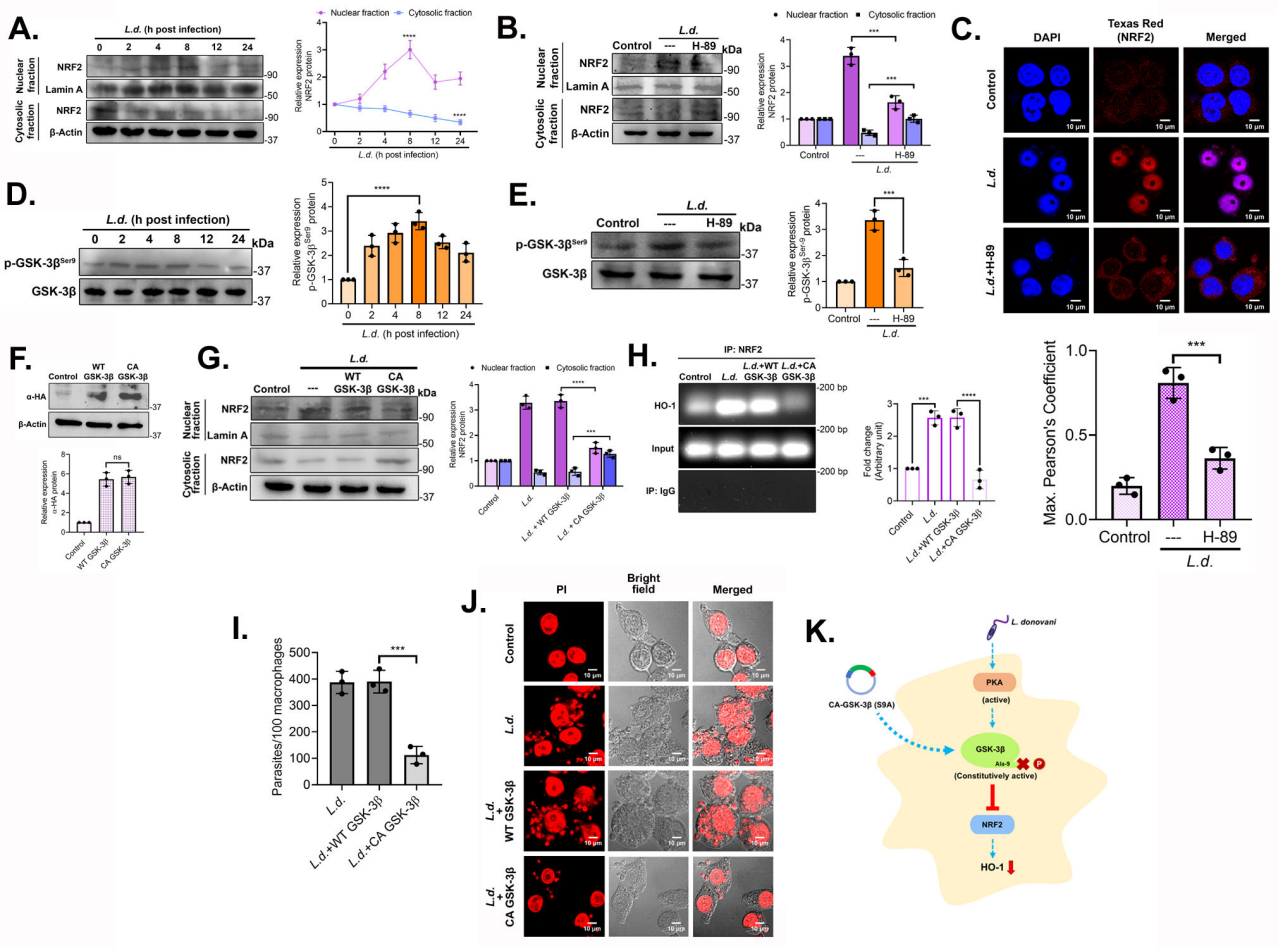
PKA facilitates NRF2 nuclear translocation by inhibiting GSK-3β activity. (**A**) RAW cells were infected with *L. donovani* at indicated periods, and the levels of NRF2 were assessed in nuclear and cytosolic fractions by immunoblotting. (**B**) RAW cells were pretreated with H-89 (10 µM) followed by *L. donovani* infection, and the levels of NRF2 were assessed in nuclear and cytosolic fractions by immunoblotting. (**C**) RAW cells were infected with *L. donovani* promastigotes for 8 h and stained with anti-NRF2 antibody followed by Texas Red-conjugated secondary antibody. Nuclei were stained with DAPI and were analyzed under a confocal microscope. Images obtained were analyzed for colocalization using ImageJ. Maximum Pearson’s Coefficient was calculated by randomly selecting at least 15 cells per field for at least three random fields per experiment using ImageJ plug-in JACoP. (**D**) RAW cells were infected with *L. donovani,* for various periods, and expression of p-GSK-3β^Ser-9^ was determined by immunoblotting. (**E**) Macrophages were pretreated with H-89 (10 µM), followed by *L. donovani* infection, and p-GSK-3β^Ser-9^ levels were determined by immunoblotting. (**F**) RAW 264.7 cells were transfected with either WT- or CA-GSK-3β expression plasmids, followed by infection with *L. donovani* for 8 h, and the expression of hemagglutinin (HA) in whole-cell lysates was analyzed by Western blotting. (**G**) Cells were transfected with WT- or CA-GSK-3β plasmids and infected with *L. donovani,* and the levels of NRF2 were determined in nuclear and cytosolic fractions by immunoblotting. (**H**) DNA from *L. donovani*–infected RAW cells was immunoprecipitated with anti-NRF2 antibody or normal immunoglobulin G. Immunoprecipitated DNA was then analyzed using HO-1 promoter-specific primers by PCR, followed by agarose gel electrophoresis. (**I and J**) BMDMs were either left uninfected or transfected with either WT- or CA-GSK-3β expression plasmids and infected with *L. donovani* promastigotes. The number of parasites per 100 macrophages was determined by PI staining (**I**), and representative confocal microscopic images are depicted (**J**). (**K**) Schematic representation of PKA-mediated inhibition of GSK-3β followed by NRF2 translocation and HO-1 expression during infection. The graph shows the combined (mean) outcomes from the indicated number of independent experiments, and the error bars indicate the variation between those independent repeats (mean ± SD); ns, not significant, ***P < 0.001; ****P < 0.0001 (ANOVA with Tukey *post hoc* test).

Apart from CREB, another known major transcription factor for HO-1 is NRF2. We earlier showed its level to be increased in the nucleus during the early hours (0–4 h) of infection (25). We therefore studied the time kinetics of p-NRF2 nuclear translocation during late hours of infection (0–24 h) and found its maximum level at 8 h post-infection (3.0-fold increase compared with uninfected control, *P* < 0.0001) (Fig. 4A). To check whether NRF2, like CREB, might as well be phosphorylated by PKA, we studied the nuclear localization of p-NRF2 in uninfected control, infected, and H-89-treated infected macrophages at 8 h post-infection. Pretreatment with H-89 showed a significant decrease in p-NRF2 in the nuclear fraction compared to the infected control (51.9% decrease compared with infected control, *P* = 0.0002) (Fig. 4B). Microscopic analysis further confirmed enhanced nuclear translocation of NRF2 during infection, which was markedly reduced upon H-89 treatment (55.1% decrease in Pearson’s coefficient compared with uninfected control, *P* = 0.0006) (Fig. 4C). Apart from KEAP, GSK-3β is another agent that sequesters NRF2 in the cytosol (36). We therefore checked the time kinetics (0–24 h) of GSK-3β and found no such significant difference in the protein level expression during infection. Contrarily, p-GSK-3β protein level was found to be maximum at 8 h post-infection (3.4-fold increase compared with uninfected control, *P* < 0.0001) (Fig. 4D). As regulation of GSK-3β activity is critically dependent on its phosphorylation at the Ser-9 position (37), we further hypothesized that *L. donovani* facilitates GSK-3β phosphorylation with the help of upstream kinase PKA. Pretreatment with H-89 markedly reduced p-GSK-3β^Ser9^ protein level at 8 h post-infection compared to the infected control (54.8% decrease compared with infected control, *P* = 0.0006) (Fig. 4E), implying the role of PKA in inhibiting GSK-3β activity. To further confirm the role of GSK-3β in inhibiting the nuclear translocation of NRF2, we transfected macrophages with constitutively active (CA)-GSK-3β construct followed by infection at an 8 h time point. Transfection efficiency was determined in wild-type (WT)-GSK-3β- or CA-GSK-3β-transfected cells by hemagglutinin (HA) expression (Fig. 4F). Transfections with either WT-GSK-3β or CA-GSK-3β did not show any detectable change in the rate of infection (data not shown). Results showed a marked reduction in nuclear NRF2 (55.1% decrease compared with WT-GSK-3β-transfected *L. donovani-*infected cells, *P* < 0.0001) (Fig. 4G). ChIP assay showed significantly reduced nuclear NRF2 binding to the HO-1 promoter in CA-GSK-3β-transfected infected cells (74.2% compared to WT-GSK-3β-transfected *L. donovani-*infected cells, *P* < 0.0001) at 8 h post-infection (Fig. 4H). Microscopic observation of parasite survival in BMDM also corroborated the previous result (71.2% decrease compared with WT GSK-3β-transfected infected cells, *P* = 0.0003) (Fig. 4I and J). All these findings suggested that *L. donovani* facilitates NRF2 translocation by inhibiting GSK-3β via PKA and induces HO-1 expression (Fig. 4K). This study, therefore, unravels the role of A_2A_R in assisting infection and thus, as a potential drug target against VL.

### Effect of ST 1535 in regulating A_2A_R-HO-1 signaling

**Fig 5:**
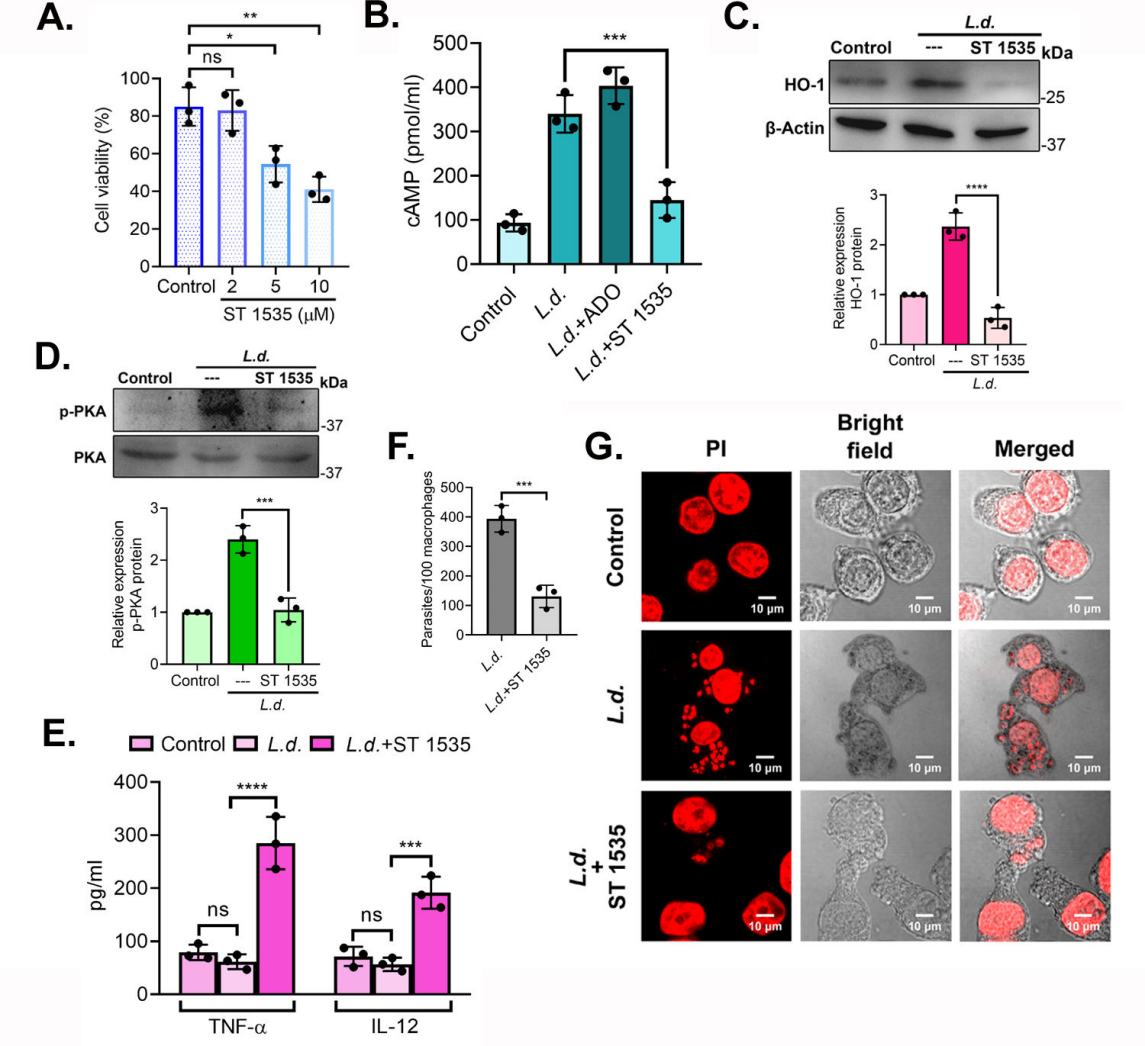
Effect of ST 1535 on A_2A_R-HO-1 signaling *in vitro*. (**A**) Macrophages were treated with ST 1535 in various doses, and cell viability was monitored by MTT assay. (**B**) Intracellular cAMP level was determined in ST 1535-pretreated (2 µM) (30 min) infected RAW cells. Adenosine (ADO) (100 µM) was used as a positive control. (**C and D**) RAW cells were pretreated with ST 1535 (2 μM) for 30 min followed by *L. donovani* infection for 8 h, and the protein level expression of HO-1 (**C**) and p-PKA (**D**) was measured by immunoblotting. (**E**) RAW cells were pretreated with LPS (100 ng/mL) and ST 1535 (2 µM) followed by *L. donovani* infection for 8 h, and cell culture supernatants were assayed for TNF-α and IL-12 using ELISA. (**F and G**) RAW cells were either uninfected or pretreated with ST 1535 and infected with *L. donovani* promastigotes for 8 h. The number of parasites per 100 macrophages was determined by PI staining (**F**), and representative confocal microscopic images are depicted (**G**). The graph shows the combined (mean) outcomes from the indicated number of independent experiments, and the error bars indicate the variation between those independent repeats (mean ± SD); ns, not significant, *P < 0.05, **P < 0.01, ***P < 0.001, and ****P < 0.0001 (Student’s *t* test and ANOVA with Tukey *post hoc* test).

ST 1535 has been identified as a preferred A_2A_R antagonist due to its prolonged pharmacodynamic effects (38). As we considered evaluating its potential as a therapeutic candidate against VL, we tested the effects of various doses of ST 1535 on RAW cell viability and identified 2 µM as the dosage with the minimal adverse impact (Fig. 5A). Next, to assess its effect in inhibiting A_2A_R activity, we checked the level of intracellular cAMP level in RAW cells. Cells pretreated with adenosine (ADO) (100 µM) (15) worked as a positive control and confirmed its role in A_2A_R activation. Pretreatment with ST 1535 (2 μM) followed by 8 h infection significantly reduced cAMP level (57.4% decrease compared with infected control, *P* = 0.0009) (Fig. 5B). Cells pretreated with ST1535 exhibited an infection pattern comparable to that of infected controls. (see Fig. S1E at https://doi.org/10.6084/m9.figshare.30597143). We further checked HO-1 protein level and found marked reduction in its expression in ST 1535-pretreated infected cells compared to infected macrophages (77.4% decrease compared with infected control, *P* < 0.0001) (Fig. 5C). Levels of p-PKA also decreased compared with those of infected cells (56.5% decrease compared with infected control, *P* = 0.0004) (Fig. 5D). Assessment of TNF-α and IL-12 showed a significant increase in ST 1535-pretreated infected cells compared to the infected control (285.2 ± 49.5 pg/mL TNF-α and 191.7 ± 30.2 pg/mL IL-12 for ST 1535, compared with 61.5 ± 14.1 pg/mL TNF-α and 56.5 ± 12.5 pg/mL IL-12 for infected control, *P* < 0.0001 and *P* = 0.0005, respectively) (Fig. 5E). ST 1535 treatment markedly reduced intracellular parasite count in RAW 264.7 cells as well (69.4% decrease compared with infected control, *P* = 0.0010) (Fig. 5F and G). All these results validated the role of ST 1535 as a potential inhibitor of A_2A_R, thus further inspiring us to check its effect on experimental VL.

### Effect of ST 1535 treatment on visceral leishmaniasis in BALB/c mice

**Fig 6:**
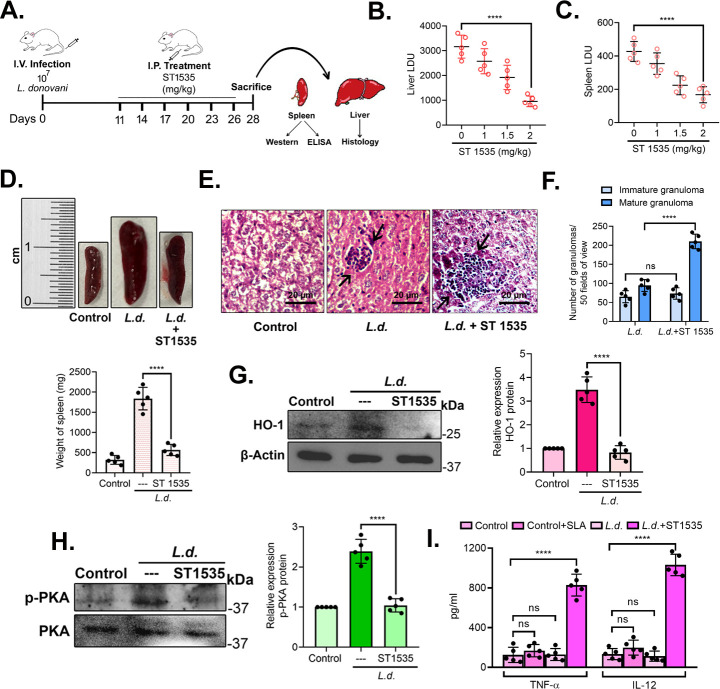
Effect of ST 1535 treatment on the progression of VL in mice. To investigate whether *L. donovani-*induced A_2A_R signaling is also operative in the *in vivo* situation, infected mice were administered intraperitoneally (i.p.) with different doses of ST 1535 ranging from 1 to 2 mg/kg body weight/day given up to 4 weeks at every 3-day interval starting at 11 days post-infection. Liver and spleen parasite burden expressed as LDU was measured at 28 days after infection. (**A**) Schematic representation of the treatment regime of ST 1535. (**B and C**) Maximum suppression of parasite burden was obtained with ST 1535 at a dose of 2 mg/kg body weight (69.8%, *P* < 0.0001) for liver (**B**) and (60.8%, *P* < 0.0001) for spleen (**C**) without causing any apparent change in the pathophysiology of the mice, and therefore this dose was chosen for the subsequent experiments. (**D**) Marked reduction in mean spleen weight was also observed in ST 1535-treated mice (69.2%, *P* < 0.0001) compared with infected ones. (**E**) Representative microscopic images of H&E-stained liver sections of uninfected control, infected, and infected + ST 1535-treated BALB/c mice showing granuloma formation (arrow). Original magnification ×40. (**F**) Total number of hepatic granulomas (immature and mature) in the H&E-stained liver sections of infected and infected + ST 1535-treated BALB/c mice were determined in 50 consecutive microscopic fields. (**G and H**) Splenocytes were isolated from uninfected control, infected, and infected + ST 1535-treated mice at 4 weeks post-infection. Splenocyte cell lysates were subjected to measurement of protein level expression of HO-1 (**G**) and p-PKA (**H**) by immunoblotting, and the cell supernatants were assayed to measure the levels of TNF-α and IL-12 by ELISA (**I**). The graph shows the combined (mean) outcomes from the indicated number of independent experiments, and the error bars indicate the variation between those independent repeats (mean ± SD); ns, not significant, ****P < 0.0001 (ANOVA with Tukey *post hoc* test).

To evaluate the importance of adenosine-A_2A_R-HO-1 signaling on parasite survival, *L. donovani-*infected BALB/c mice (*n* = 5) were administered intraperitoneally (i.p.) with various doses of ST1535 (1, 1.5, and 2 mg/kg body weight/day) (39) up to 4 weeks at 3-day intervals starting at 11 days post-infection (Fig. 6A). During the experimental period, all the animals remained healthy, and weekly monitoring revealed no observable signs of toxicity such as weakness, lethargy, or significant weight loss in all groups of mice. We also observed that the animals in the ST 1535-treated groups were active and healthy throughout the duration of the trial. The infection was allowed to proceed for 4 weeks, after which the animals were sacrificed, and the anti-leishmanial potency was assessed in terms of parasite burden in the liver and spleen as Leishman-Donovan unit (LDU) (40). Assessment of LDU showed the most significant decrease in organ parasite burden at 2 mg/kg dosage (69.8% and 60.8% decrease in liver and spleen at 4 weeks post-infection, compared with infected control, *P* < 0.0001 and *P* < 0.0001, respectively) (Fig. 6B and C); therefore, it was selected as the optimum dose, and subsequent experiments were carried out using 2 mg/kg, keeping the treatment regime same as in Fig. 6A. A marked reduction was also observed in mean spleen weight in ST 1535-treated infected mice (69.2% decrease compared with infected mice, *P* < 0.0001) (Fig. 6D). Hematoxylin and eosin staining of sections of liver from ST1535-treated infected mice at 4 weeks revealed significant granuloma formation, denoting macrophage infiltration, compared with untreated infected mice (Fig. 6E). The number of hepatic mature granulomas was also found to be significantly increased in sections from ST 1535-treated infected mice at 4 weeks post-infection in comparison to untreated infected mice (2.2-fold increase compared with infected mice, *P* < 0.0001) (Fig. 6F). HO-1 expression was determined in isolated splenocytes from uninfected control, infected, and ST1535-treated infected mice after 4 weeks post-infection. The protein level of HO-1 markedly decreased in ST1535-treated infected splenocytes (77.6% decrease compared with untreated infected splenocytes, *P* < 0.0001) (Fig. 6G). The protein level of p-PKA also decreased significantly (54.6% decrease compared with untreated infected splenocytes, *P* < 0.0001) (Fig. 6H). Next, TNF-α and IL-12 levels were assessed in supernatants of splenocytes from uninfected control, SLA-treated control, and infected and ST1535-treated infected groups to evaluate cytokine responses. In the case of ST 1535 treatment, TNF-α and IL-12 levels were markedly increased compared with infected cells (827.9 ± 110.2 pg/mL TNF-α and 1,031.1 ± 108.3 pg/mL IL-12, compared with 128.9 ± 60.7 pg/mL TNF-α and 112.4 ± 51.7 pg/mL IL-12 from infected control, *P* < 0.0001 and *P* < 0.0001, respectively) (Fig. 6I). All these results validated our previous *in vitro* findings that *L. donovani* utilizes the one-way signaling axis of adenosine-A_2A_R to induce HO-1 expression, which results in reduction of pro-inflammatory cytokine levels, thus safeguarding its survival and sustenance in the host.

## DISCUSSION

**Fig 7:**
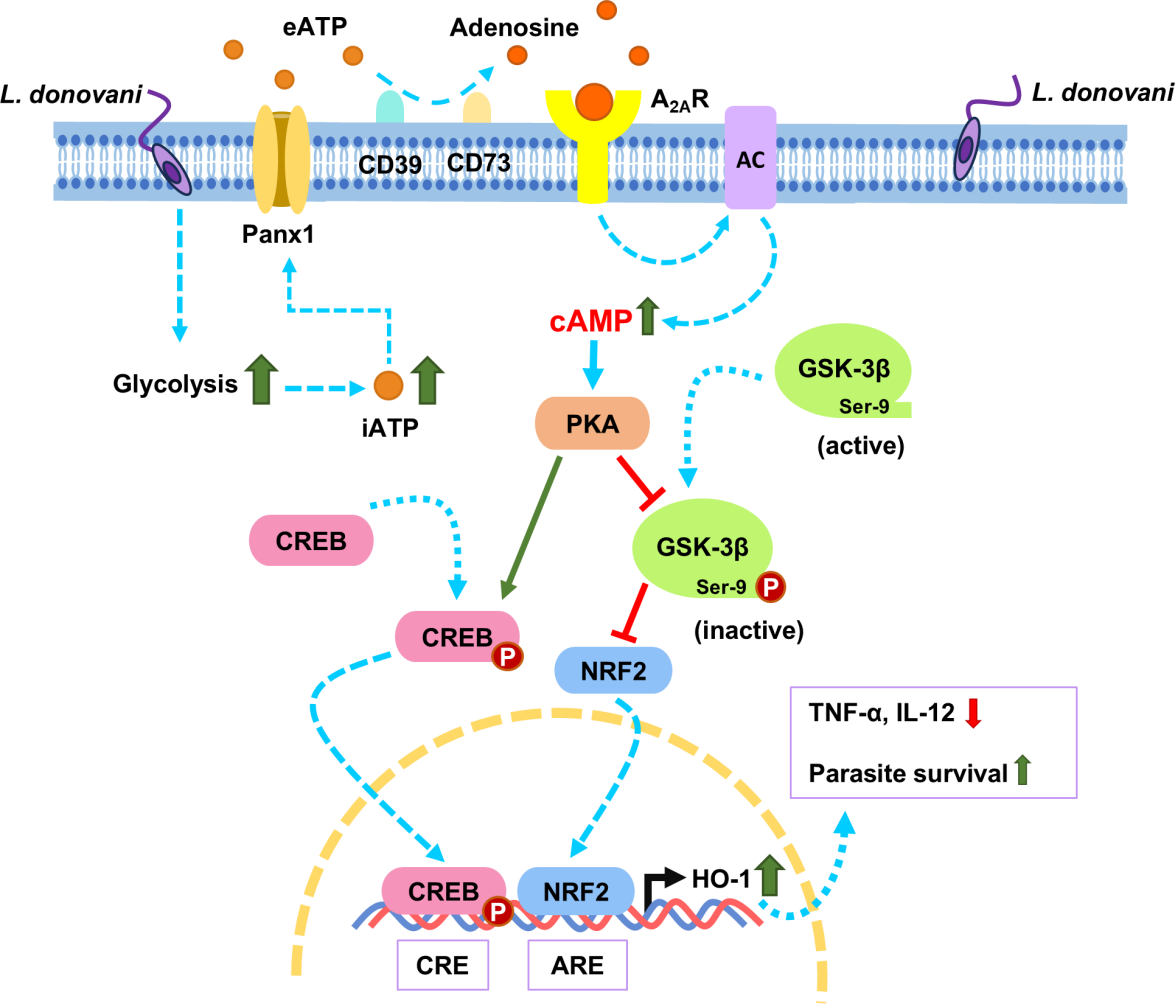
*L. donovani* induces adenosine-A_2A_R-cAMP-PKA-mediated expression of HO-1 for sustained infection. *Leishmania* elevates extracellular adenosine levels by enhancing glycolysis and releasing ATP, which subsequently activates A_2A_R (15). Adenosine-A_2A_R interaction leads to A_2A_R (a GPCR)-mediated activation of adenylate cyclase (AC), which increases intracellular cAMP levels. This elevated cAMP activates PKA, which through phosphorylation activates CREB on one hand, leading to p-CREB-mediated HO-1 induction. On the other hand, PKA inhibits GSK-3β by phosphorylation on its Ser-9 residue, thus removing its inhibitory effect over NRF2, causing its nuclear localization and NRF2-mediated induction of HO-1. This schematic diagram depicts A_2A_R-cAMP-PKA-mediated activation of HO-1 expression, resulting in the reduction of pro-inflammatory cytokines TNF-α and IL-12, facilitating parasite survival.

Previously reported as a “retaliatory metabolite” for its timely expression during cellular injury or stress, adenosine is known for its impact on immune or inflammatory cell functions through its specific receptors (41–43). Despite its predominant effect on the immune system, its overexpression and enhanced signaling create an immunosuppressed niche which then becomes a suitable environment for pathogens, causing neoplasia and infection (44–46). Intracellular adenosine released out in the extracellular space promotes parasite burden through activating A_2A_R and A_2B_R receptors during *L. infantum* infection (47), and inhibition of adenosine-mediated signaling is being discussed as a clever strategy to develop immunotherapy against cancer (48). Our previous findings regarding the antioxidant enzyme HO-1 connect its expression with adenosine-A_2A_R signaling (15, 24, 25). The idea of HO-1 being one of the expression targets of the A_2A_R signaling axis is convincing since both the effect of adenosine-A_2A_R mediated signaling and HO-1 enzymatic activity is the establishment of an anti-inflammatory environment inside the host cell macrophage that supports the survival of the parasites. Our initial experimental findings regarding inhibitor treatment of both A_2A_R and HO-1 reveal a one-way signaling axis from A_2A_R to HO-1. Since A_2A_R is a GPCR, we hypothesized that the signaling is probably conveyed by the activation of adenylate cyclase and subsequent increased production of cAMP. Induction of cAMP production has been mentioned in sepsis and marked as proof of immunometabolic modulations during infection (49). The importance of cAMP-mediated signaling in *Mycobacterium tuberculosis* is also being documented due to its positive effect on the bacteria’s potency in infection (50). With the focus on the elevated cAMP levels in a variety of diseases comes the whereabouts of cAMP-dependent effector proteins that convey the signaling message downstream. Two main cAMP-dependent effector proteins, PKA and EPAC, are extensively researched for their role in channeling GPCR signal messages (51). In many physiological scenarios when it comes to cAMP-mediated signaling, both PKA and EPAC are found to actively play their role, such as in vasorelaxation (52), chronic obstructive pulmonary diseases (53), cancer (54), and tumor microenvironment (55). In some cases of diseases and infections, the cAMP-mediated signaling may be beneficial, while in other cases, this pathway may worsen the diseases (56, 57). PKA is also documented to have an important role in the process of proliferation and differentiation of promastigotes in the host cell macrophages during *L. amazonensis* infection (58). While both PKA and EPAC-mediated signaling have strong roles to play in certain scenarios, we chose to move forward with the finding of downstream signaling from PKA, as in the case of HO-1 induction by adenosine-A_2A_R signaling, PKA was found to have more importance than EPAC. As a well-known target of PKA, CREB caught our interest since the HO-1 promoter site does have a CRE-binding element where p-CREB, the activated form of the protein, can potentially bind and is supported and proved by several studies (56, 59, 60). The question was whether this p-CREB/HO-1 binding and induction of HO-1 was due to the A_2A_R-mediated cAMP-PKA signaling axis or not, which is proved to be true in the present study. However, inhibiting CREB with siRNA treatment did not absolutely abolish HO-1 expression, thus pointing out that there must be some other transcription factors. A well-known transcription factor of HO-1, NRF2, was thus under focus. In our previous study, we already showed that NRF2 is usually inhibited by KEAP, which is elevated during *L. donovani* infection (25). A literary study revealed that NRF2 can be inhibited from translocating into the nucleus not just by the KEAP-dependent method but also by GSK-3β. While KEAP binds to the Neh2 domain of NRF2, GSK-3β phosphorylates the serine cluster in the Neh6 domain, thus targeting NRF2 for SCF-/β-TrCP-dependent degradation (57). Inhibition of GSK3 enhances NRF2 protein stability, nuclear translocation, and target gene expression in pancreatic beta cells (61). Inhibition of GSK-3β was found to be therapeutic for type 2 diabetic glomerular injury as it allows NRF2 expression and subsequent suppression of oxidative stress, thus resulting in an improvement in podocyte injury and senescence (62). As *L. donovani* significantly increases HO-1 expression, for which NRF2 plays a major role, GSK-3β is then supposed to be inhibited. One of our previous studies focused on a different branch of GSK-3β and found it to be inhibited during *L. donovani* infection through phosphorylation at its Ser-9 residue (37). Inducing GSK-3β Ser-9 phosphorylation through AMPK, a kinase, liberates NRF2 and improves cognitive impairment and pathological features in Alzheimer’s disease (63). Other studies highlight PKA being another kinase for phosphorylating GSK-3β and inhibiting it (64, 65). These findings helped us connect the dots that GSK-3β can potentially be phosphorylated by upstream kinase PKA, resulting in the liberation of its inhibitory effect on NRF2 and regulating HO-1 expression. However, there is another kinase called AKT that is documented to have a similar role in the regulation and inhibition of GSK-3β during VL (37). Hence, it may be anticipated that activation of downstream transcription factors and subsequent HO-1 activation may not solely be a result of adenosine-A_2A_R activation of PKA. However, further studies are necessary to determine whether A_2A_R signaling can regulate AKT-mediated regulation or not. In this study, inhibition of PKA revealed a marked reduction of GSK-3β^Ser9^ and subsequent nuclear localization of NRF2 and induction of HO-1. All these findings finally cleared the image of a detailed signaling axis, starting from adenosine-mediated A_2A_R activation, leading to cAMP-PKA-NRF-/CREB-mediated induction of HO-1 expression, which ultimately results in reducing pro-inflammation and supporting parasite survival (Fig. 7).

Although this study highlights important insights into the immunometabolic functions and therapeutic strategies during VL, some limitations should be noted. The murine model used in this study provided a valuable understanding of host–parasite interactions; however, it does not fully reflect the chronic, progressive, and fatal nature of the disease seen in humans as murine models generally exhibit a self-healing infection. Despite this, the results offer a solid experimental basis for understanding key mechanisms of infection. Future studies using human samples will be important to confirm these findings and strengthen their relevance to human disease.

## MATERIALS AND METHODS

### Reagents

Most of the reagents used in the experiments are listed in Table 1. For others, the source has been mentioned along with the methodology.

**TABLE 1:** List of chemical compounds used in experiments

Reagent	Source	Identifier/RRID	Dilution or concentration	
Antibodies				
Adenosine A2aR Antibody	Novus Biologicals	7F6-G5-A2	1:1,000	
Heme Oxygenase 1/HMOX1 Antibody (A-3)	Santa Cruz Biotechnology	sc-136960	1:1,000	
Phospho-PKA C (Thr197) Antibody	Cell Signaling Technology	4781	1:1,000	
PKA C-α Antibody	Cell Signaling Technology	4782	1:1,000	
CREB1 Antibody (X-12)	Santa Cruz Biotechnology	sc-240	1:1,000	
p-CREB-1 Antibody (Ser 133)	Santa Cruz Biotechnology	sc-101663	1:1,000	
Nrf2 Antibody (H-10)	Santa Cruz Biotechnology	sc-518036	1:1,000	
HIF1A Polyclonal Antibody	Thermo Fisher Scientific	PA1-16601	1:1,000	
GSK3B Monoclonal Antibody (ZG004)	Thermo Fisher Scientific	39-9500	1:1,000	
Phospho-GSK3B (Ser9) Monoclonal Antibody (C.367.3)	Thermo Fisher Scientific	MA5-14873	1:1,000	
Anti-Lamin A antibody [133A2]	Abcam	ab8980	1:1,000	
Purified anti-HA.11 Epitope Tag Antibody	BioLegend	901502	1:2,000	
Goat Anti-Mouse IgG H&L (Texas Red)	Abcam	ab6787	1:500	
Anti-Mouse IgG (whole molecule)–peroxidase antibody produced in goat	Sigma-Aldrich	A4416	1:10,000	
Anti-Rabbit IgG (whole molecule)–peroxidase antibody produced in goat	Sigma-Aldrich	A0545	1:10,000	
Anti-Goat IgG (whole molecule)–peroxidase antibody produced in rabbit	Sigma-Aldrich	A5420	1:10,000	
Chemicals				
ZM 241385	Sigma-Aldrich	Z0153	1 µM	
Tin Protoporphyrin IX dichloride (SnPP)	Santa Cruz Biotechnology	14325-05-4	20 µM	
2’ ,5′-Dideoxyadenosine (adenylate cyclase inhibitor)	Sigma-Aldrich	D7408	100 µM	
H-89 dihydrochloride (protein Kinase A inhibitor)	Sigma-Aldrich	B1427	10 µM	
ESI-09 (EPAC inhibitor)	Sigma-Aldrich	5.00506.0001	10 µM	
Adenosine	Sigma-Aldrich	A9251	100 µM	
Lipopolysaccharides from *Escherichia coli* O111:B4	Sigma-Aldrich	L2630	100 ng/mL	
siRNAs				
CREB1 siRNA (m)	Santa Cruz Biotechnology	sc-35111		
Control siRNA-A	Santa Cruz Biotechnology	sc-37007		
Commercial kits				
RNeasy Mini Kit	Qiagen	74104		
Mouse TNF alpha ELISA Kit	Abcam	ab100747		
Mouse IL-12 p40 + IL-12 p70 ELISA Kit	Abcam	ab100699		
Cyclic AMP ELISA Kit	Cayman Chemical	581001		
Imprint Chromatin Immunoprecipitation Kit Protocol	Merck	CHP1		

### Cells and parasites

*L. donovani* strain MHOM/IN/1983/AG83 was isolated from an Indian Kala-azar patient (66) and maintained as described earlier (25). The murine macrophage cell line RAW 264.7 (National Repository for Cell Lines/Hybridomas, Department of Biotechnology, Government of India) was maintained at 37°C, 5% CO_2_ in Roswell Park Memorial Institute Medium (RPMI) 1640 (Invitrogen) supplemented with 10% FBS, penicillin (100 U/mL), and streptomycin (100 µg/mL). *In vitro* infection of macrophages was carried out with *L. donovani* promastigotes at a parasite/cell ratio of 10:1 (24) for specific periods of incubation.

### Cytokine analysis by ELISA

TNF-α and IL-12p70 cytokine levels were measured in cell supernatants from RAW 264.7 cells and mouse splenocytes, as instructed in the manufacturer’s protocol.

### Real-time PCR

Total RNA from RAW 264.7 cells was extracted using the RNeasy mini kit (Qiagen) as per the manufacturer’s instructions, and the RNA (1 µg) was reverse-transcribed using the SuperScript First-Strand synthesis system for the RT-PCR kit (Invitrogen). The synthesized cDNA was used for real-time PCR analysis using SYBR Green JumpStart Taq ReadyMix for quantitative PCR, capillary formulation (Sigma-Aldrich) on LightCycler 96 instrument (Roche). The PCR amplification conditions that were maintained throughout the whole amplification process were 40 cycles of 95°C for 10 sec and 72°C for 11 sec. β-Actin mRNA was used as an endogenous control to normalize target mRNA values, and data were expressed relative to normalized values of the corresponding controls using the delta delta Ct method (2^−ΔΔCt^), where Ct is the threshold cycle, at which point the fluorescence above the background is detectable. Oligonucleotides used for real-time PCR were as follows: for β-actin, 5ʹ-TTGTGATGGACTCCGGAGAC-3ʹ (F) and 5ʹ-TGATGTCACGCACGATTTCC-3ʹ (R); for HO-1, 5ʹ-CATGCCCCAGGATTTGTCTG-3ʹ (F) and 5ʹ-CAGGGCCGTGTAGATATGGT-3ʹ (R).

### Cytotoxicity assay

MTT assay was performed to monitor the effect of ST 1535 on RAW 264.7 cell viability. A total of 1 × 10^4^ cells were grown in a 96-well plate and incubated overnight. The cells were treated with respective different concentrations of ST 1535 (2, 5, and 10 µM) and incubated for 4 h. MTT (5 mg/mL) was then added and incubated at 37°C for 4 h. Thereafter, formazan crystals were solubilized in a solubilization buffer, and absorbance was measured at 570 nm. The extent of cell viability was measured as the percentage of viability in comparison with the untreated cells.

### Measurement of heme content

For intracellular heme content assessment, cells were cultured, and after treatment, the cells were washed twice with 1 × PBS, and an assay was performed 2 h later. Heme content was determined according to the method of Motterlini et al. (67). Briefly, cells were washed with PBS and solubilized by adding 1 mL of concentrated formic acid. The heme concentration of the formic acid solution was determined spectrophotometrically at 398 nm (extinction coefficient 1.56 × 10^5^ M^−1^ cm^−1^). Heme content was expressed as picomoles per mL.

### cAMP assay

The intracellular cAMP level was measured by using a cAMP assay kit from Cayman Chemical according to the manufacturer’s protocol.

### RNA-mediated interference by siRNA transfection

Transfection was carried out with control/specific siRNAs (Table 1). RAW 264.7 cells were plated in tissue culture plates at a density of 2 × 10^6^ cells/plate in antibiotic-free and serum-free RPMI, followed by transfection with siRNAs as per the manufacturer’s instructions. Following transfection, the knockdown efficiency was verified by Western blotting. siRNA with comparatively higher knockdown efficiency was utilized for the study.

### Plasmid transfection

We thank Dr. Jim Woodgett (senior investigator, Lunenfeld-Tanenbaum Research Institute) for gifting us WT-GSK-3β (HA GSK-3β WT pcDNA3) and CA-GSK-3β (HA-GSK-3β S9A pcDNA3) (Addgene plasmid # 14753 and Addgene Plasmid # 14754, respectively). RAW 264.7 cells were plated in tissue culture plates at a density of 2 × 10^6^ cells/plate in antibiotic-free and serum-free RPMI, followed by transfection with plasmids, as previously mentioned (68, 69).

### Immunoblotting

After indicated treatments and infections, cells were lysed using a cocktail of 1 × ice cold lysis buffer (Cell Signaling Technology) supplemented with phenylmethylsulfonyl fluoride (PMSF) and protease inhibitor cocktail (PIC), and the protein concentrations in the cell lysates were estimated using the Bradford assay (70). Fifty micrograms of protein was then resolved by 10% SDS-PAGE and then transferred to a nitrocellulose membrane (Millipore). Also, 5% bovine serum albumin in Tris-buffered saline solution was used for blocking the membrane, which was then incubated with primary antibody overnight at a dilution recommended by the manufacturer. After washing with wash buffer (Tris-buffered saline-T), membranes were probed with horseradish peroxidase-conjugated secondary antibody for 1 h, washed again with TBST, and detected by chemiluminescence using ECL solution (Bio-Rad). Quantification of band intensities was conducted using the ImageJ software. β-Actin was used as a loading control.

### Isolation of the nuclear fraction

To prepare subcellular fractions, the cells were washed twice with ice-cold 1 × PBS and centrifuged at 4°C for 10 min at 8,000 rpm to obtain the cell pellet. The cells were then lysed through resuspending in 5 volumes of hypotonic buffer A (10 mM HEPES [pH 7.9], 10 mM KCl, 0.1 mM EDTA, 0.3% NP40, 0.5 mM PMSF, 10 mg of leupeptin per mL, 10 mg of pepstatin per mL, and 0.01 U of aprotinin per mL). The solution was incubated on ice for 5 minutes and vortexed from time to time, followed by homogenization using a narrow-gage syringe. The solution was then centrifuged at 4°C for 10 min at 10,000 × *g,* and the supernatant was collected as the cytosolic extract. The pellet was washed twice with ice-cold buffer A without NP40, and the pellet was resuspended in equal volumes of buffer B (20 mM HEPES [pH 7.9], 0.4 M NaCl, 1 mM EDTA, 0.5 mM PMSF, 10 mg of leupeptin per mL, 10 mg of pepstatin per mL, 0.01 U of aprotinin per mL, and 25% glycerol). The solution was incubated on ice for 10 min and vortexed from time to time. After centrifugation at 4°C for 10 min at 14,000 *g*, the supernatant solution representing the nuclear fraction was isolated.

### Confocal microscopy

Macrophages were plated onto coverslips and cultured overnight. The cells were treated as required and infected with *L. donovani* promastigotes for the indicated time point. Cells were then washed twice in 1 × chilled PBS, fixed and permeabilized with chilled methanol in −20°C for 3 minutes, incubated with 0.1% Triton X-100 in 2% BSA blocking solution for further permeabilization for 30 minutes, followed by primary antibody overnight at 4°C. After washing with PBS, coverslips were incubated with fluorescent dye–conjugated secondary antibodies for 1 h at room temperature. The cells were stained with 4ʹ,6-diamidino-2-phenylindole (DAPI) (1 µg/mL) in 1 × PBS plus 10 µg/mL RNase A to label the nucleus, mounted on slides, and visualized under a confocal microscope (Carl Zeiss) using 63× oil immersion objective. Images obtained were analyzed by Image J software (https://imagej.net/ij/) using the plug-in JACoP (71).

### ChIP assay

Cells were cross-linked with 1% formaldehyde, harvested in lysis buffer (1% SDS, 10 mM EDTA, 50 mM Tris–HCl, pH 8.0, and 1 × protease inhibitor mixture), and sonicated, followed by immunoprecipitation with antibodies. Immunoprecipitation with a normal rabbit IgG served as a negative control. Immunoprecipitated cell lysates were incubated with protein A/G plus agarose, washed, and then heated at 65°C for 1.5 h to reverse the cross-linking. DNA fragments were purified, and PCR amplification was performed using 5 µL of DNA (recovered from ChIP) with 35 cycles of denaturation, annealing, and extension, and amplified PCR products were analyzed by electrophoresis on a 1% agarose gel. The following primer pairs were used to amplify putative HO-1 promoter regions, 5ʹ-GAGAAGGCAGCCAAACACTC-3ʹ (sense) and 5ʹ-GGTTTGCTGACACTTGCCTT-3ʹ (antisense).

### Assessment of intracellular infection

For *in vitro* experiments, cells were plated on coverslips placed in tissue culture plates. Cells were then infected with *L. donovani* promastigotes and incubated for 8 h. After incubation, cells were fixed with methanol and stained with PI (1 µg/mL; Sigma) in 1 × PBS along with 10 µg/mL RNaseA (72). At the end of the assay, the number of parasites was determined by observing under a confocal microscope (Carl Zeiss and Olympus). Images obtained were analyzed by ImageJ software.

### BMDM isolation

BMDMs were isolated from the femurs and tibiae of euthanized BALB/c mice (6–8 weeks old) (73), incubated in the media in the presence of GM-CSF (74), treated, and infected with promastigotes as required.

### *In vivo* infection

Animal maintenance and experiments were performed following the guidelines provided by the Committee for Control and Supervision of Experiments on Animals. The protocol was approved by the Departmental Animal Ethics Committee (Institutional Animal Ethics Committee, Department of Biochemistry, University of Calcutta). For *in vivo* infection, 6- or 8-week-old female BALB/c mice (~20 g) were maintained in a temperature-controlled environment with a 12 h light/12 h dark cycle and provided with a standard diet and water *ad libitum*. Mice were kept in a pathogen-free room at the animal house of the institute for more than 1 week before experimental infection. All procedures were performed according to the protocol approved by the Institutional Animal Ethics Committee. Mice were injected via the tail vein with 1 × 10^7^ stationary-phase *L. donovani* promastigotes as described earlier (24). On the 11th day post-infection, mice were intraperitoneally administered with ST 1535 at doses of 1, 1.5, and 2 mg/kg body weight/day every 3rd day till 26 days post-infection. On the 28th day, the mice were euthanized, and infection was assessed by removing the liver and spleen from infected mice. Liver and spleen weights were monitored using an electronic precision balance. Parasite burden was determined from Giemsa-stained impression smears (75). Liver and spleen parasite burdens, expressed as LDU, were calculated as the number of amastigotes/1,000 nucleated cells × organ wt (in grams) (40). Throughout the experimental time, animals were checked for body weight, activity, and body temperature. Splenocytes from BALB/c mice were isolated and cultured (1 × 10⁶ cells/mL) as described previously. Soluble *Leishmania* antigen (SLA) was prepared as described earlier (73), and 20 µg/mL of SLA was used to stimulate the adherent splenocytes for 48 h. The supernatant was used for measuring cytokine levels through ELISA, while the cells were used for protein expression analysis through immunoblotting.

### Histopathology

Isolated livers were fixed in 10% formalin (Merck) and embedded in paraffin wax. Tissue sections (5 mm) were made with a microtome (Leica Biosystems) and stained with H&E to study their microarchitecture by light microscopy (76).

### Densitometric analysis

Densitometric analysis for all the experiments was carried out using ImageJ software. Band intensities were quantitated densitometrically, and the values obtained were normalized to endogenous control and expressed in arbitrary densitometric units, as indicated in bar graphs adjacent to the figures.

### Statistical analysis

Data shown are representative of at least three independent experiments unless otherwise stated as *n* values given in the legend. For *in vitro* studies, macrophage cultures were prepared in biological triplicates (*n* = 3), while *in vivo* experiments were performed using splenocytes from five individual animals per group (*n* = 5). The results are expressed as mean ± SD from the indicated number of experiments. Statistical analysis was carried out using GraphPad Prism 8.0.1 Software. For comparison between two groups, Student’s *t*-test was used, and for comparison between three or more groups, one-way ANOVA with the Tukey *post hoc* test was used. To assess the statistical differences among pairs of data sets, a *P* value of < 0.05 is considered to be significant. Western blot quantitation was performed using ImageJ software.

## Data Availability

All relevant data can be found within the article.
